# Chronic Use of Statins and Their Effect on Prevention of Post-Endoscopic Retrograde Cholangiopancreatography Pancreatitis

**DOI:** 10.3389/fphar.2018.00704

**Published:** 2018-06-29

**Authors:** Mahmud Mahamid, Abdulla Watad, Nicola L. Bragazzi, Dov Wengrower, Julie Wolff, Dan Livovsky, Howard Amital, Mohammad Adawi, Eran Goldin

**Affiliations:** ^1^Disgestive Diseases Institute Sharee Zedek Medical Center, Jerusalem, Israel; ^2^Endoscopy Unit, Faculty of Medicine, Nazareth Hospital EMMS Bar-Ilan University, Safed, Israel; ^3^Department of Medicine 'B', The Zabludowicz Center for Autoimmune Diseases Sheba Medical Center, Tel-Hashomer, Israel; ^4^Sackler Faculty of Medicine Tel-Aviv University, Tel-Aviv, Israel; ^5^Department of Health Sciences, School of Public Health University of Genoa, Genoa, Italy; ^6^Department of Rehabilitation Sheba Medical Center, Tel-Hashomer, Israel; ^7^Faculty of Medicine, Ziv and Padeh Hospitals Bar-Ilan University, Safed, Israel

**Keywords:** endoscopic retrograde cholangiopancreatography (ERCP), hyperamylasemia, pancreatitis, statins, endoscopy

## Abstract

**Background and Aims:** Post-endoscopic retrograde cholangiopancreatography (ERCP) pancreatitis (PEP) is one of the major complications of ERCP. Thus, several non-invasive as well as invasive strategies have been investigated as preventative therapies for PEP with various efficacy.

**Methods:** We enrolled any patients who underwent ERCP both at the Shaare Zedek Medical Center in Jerusalem and EMMS Nazareth hospital. Association between use of statins and different variables were assessed with univariate tests (chi-squared for categorical variables). Predictors of incidence of PEP and severity of pancreatitis were computed using conditional logistic regression, correcting for potential confounding factors.

**Results:** 958 subjects were analyzed. Average age was 62.04 ± 21.18 years (median 68 years). Most of the patients were female (*n* = 558, 58.2%), Jewish (*n* = 827, 86.3%), and inpatients (*n* = 631, 65.9%). Only few ERCPs were performed emergently (*n* = 40, 4.2%). Twenty-Seven patients repeated the exam. Overall incidence of PEP/hyperamylasemia was 16.8% (*n* = 161); with a 5.6% (*n* = 54) incidence of hyperamylasemia and a 11.2% (*n* = 107) incidence of pancreatitis. Overall, 6 cases of severe pancreatitis were identified. The logistic regression analysis demonstrated that chronic use of statins is a protective factor in preventing development of PEP/hyperamylasemia [OR 0.436 [95%CI 0.303–0.627], *p* < 0.001]; Particularly, the PEP OR was of 0.318 [95%CI 0.169–0.597], *p* < 0.001 and the hyperamylasemia OR was of 0.565 [95%CI 0.372–0.859], *p* = 0.008. No significant predictor could be found for the risk of developing severe PEP.

**Conclusions:** Our data support the possibility of exploiting statins as preventive agents for PEP. However, further studies, mainly RCTs, are warranted in order to replicate our findings.

## Introduction

Endoscopic retrograde cholangiopancreatography (ERCP) is the current gold-standard for the diagnosis and treatment of pancreatic and biliary diseases (Canlas and Branch, 2007). It is normally used for stone disease, ampulla of Vater/papillary abnormalities, and biliary and pancreatic ductal disorders (Bor et al., 2015; Ahmed et al., 2017). Nevertheless, ERCP use has decreased in the last decade due to the widespread availability of new, non-invasive imaging modalities such as magnetic resonance cholangiopancreatography (MRCP), CT and endoscopic US (Ahmed et al., 2017). Post-ERCP pancreatitis (PEP) may arise as a complication, together with bleeding, perforation, and cholangitis, with an incidence as high as 15–20% among high-risk cases (Canlas and Branch, 2007; Chen et al., 2014). PEP consists of new or worsening abdominal pain, associated with high serum levels of amylase (at least three times the upper limit of normal) with an onset of more than 24 h after the procedure (Dumonceau et al., 2014). The major risk factors for PEP include: female gender, previous pancreatitis, previous PEP, sphincter of Oddi dysfunction, intraductal papillary mucinous neoplasm, difficult cannulation, endoscopic sphincterotomy (EST), precut sphincterotomy, and main pancreatic duct injection (Ding et al., 2015; Wang, 2017). As such, prevention modalities for PEP including patient selection, risk-stratification and/or prophylactic pancreatic stent placement, are of fundamental importance (Tarnasky et al., 1998; Wong and Tsai, 2014). Concerning pharmaco-prevention modalities, to date, non-steroidal anti-inflammatory drugs (NSAIDs) are the only medications to be effective in PEP prevention (Cote and Elmunzer, 2016; Chandrasekhara et al., 2017). Furthermore, evidence is emerging that rectal administration of NSAIDs such as indomethacin or diclofenac just before or after ERCP might reduce the risk and severity of post-procedure pancreatitis (Dumonceau et al., 2014; Qian et al., 2017). According to the updated European Society of Gastrointestinal Endoscopy (ESGE) guidelines for PEP prevention, there is no evidence that drugs such as corticosteroids, antioxidants, heparin, sphincter of Oddi pressure reducers, and/or anti-inflammatory drugs (other than indomethacin and diclofenac) can effectively reduce the risk of PEP (Dumonceau et al., 2014).

Statins, also known as 3-Hydroxy-3-methylglutaryl-coenzyme A (HMG-CoA) reductase inhibitors, are commonly used for lowering blood lipid levels and reducing cardiovascular risk (Schachter, 2005). Several studies have reported statins as a cause of acute pancreatitis (Badalov et al., 2007), yet, many others have shown their efficacy in preventing it (de-Madaria, 2017). Interestingly, preliminary results show that chronic statin administration appears to have a protective effect on the development of PEP (Martinez-Moneo et al., 2017), yet there are very few studies to assess such findings. The aim of our study was thus, to fill this gap in knowledge.

## Materials and methods

### Ethics statement

The current study received ethical approval from the hospital ethical committee and was conducted according to the Helsinki guidelines. The data was coded to preserve the anonymity of the patients. Informed consent was waived because of the non-interventional study design.

### Patients selection

All patients who underwent for ERCP at either the Shaare Zedek Medical Center in Jerusalem or EMMS Nazareth hospital (Israel) within the study period of 2013–2015 were considered potentially eligible and, as such, were enrolled in the study. All patients underwent to ERCP due to common bile duct (CBD) disorders and none of them had pancreatic duct or sphincter of Oddi manometry studies.

PEP was defined as onset of abdominal pain more than 24 h after the procedure, lasting more than 4 h, with high serum levels of amylase at least three times the upper limit of normal and patient admission required. Hyperamylasemia was defined as an increase of serum levels of amylase above the upper limit of normal range (normal value 0-90 U/L), associated with abdominal pain of a duration of less than 4 h. Severity of pancreatitis was assessed using both revised Atlanta criteria and APACHE II scores (Larvin and McMahon, 1989; Banks et al., 2013).

Consumers of statins were defined as patients taking statins for a period of at least 6 months before the ERCP procedure. Patients were treated with 20–40 mg/die of Simvastatin. Ethnicity was classified as Jew and non-Jew based on surname analysis. ERCP procedure was also classified as in an inpatient and outpatient procedure.

### Statistical analyses

Data was retrieved from medical charts, extracted and entered in an *ad hoc* pre-designed spreadsheet. All data was reviewed by an expert gastroenterologist. Before starting any statistical analysis, data was visually inspected and checked for outliers. Normality of data distribution was checked with the Kolmogorov-Smirnov test. Continuous variables were computed as mean ± standard deviation and median, whilst categorical variables were expressed as percentages. The association between the use of statins and different variables was assessed with univariate tests (chi-squared for categorical variables). Predictors of incidence of PEP and severity of pancreatitis were computed using conditional logistic regression, correcting for potential confounding factors. All statistical analyses were carried out with a commercial software, Statistical Package for Social Science (SPSS version 24.0, IBM, Chicago, IL, USA). Figures with *p*-value less than 0.05 were considered statistically significant.

## Results

Of an initial list of 987 patients, after removing duplicates, 958 subjects (97.1% of the original sample) were analyzed. Average age was 62.04 ± 21.18 years (median 68 years). Most of the patients were female (*n* = 558, 58.2%), Jewish (*n* = 827, 86.3%), and inpatients (*n* = 631, 65.9%). Only few ERCPs were performed emergently (*n* = 40, 4.2%). Twenty-Seven patients repeated the exam (mean age 65.67 ± 19.08, median 70): most of them were Jewish (*n* = 22, 81.5%), female (*n* = 18, 66.7%), outpatients (*n* = 14, 51.9%), and consumers of statins (*n* = 15, 55.6%).

Overall incidence of PEP/hyperamylasemia was 16.8% (*n* = 161): more specifically, incidence of hyperamylasemia and of pancreatitis was 5.6% (*n* = 54), and 11.2% (*n* = 107), respectively. Overall, 6 cases of severe pancreatitis were identified. More details are shown in Table 1.

**Table 1:** Characteristics of the study population.

**Variable**	**Value**
Age (mean; median)	62.04 ± 21.18; 68
GENDER (n; %)	
Male	400 (41.8%)
Female	558 (58.2%)
ETHNICITY (n; %)	
Non-Jew	131 (13.7%)
Jew	827 (86.3%)
Inpatient (n; %)	631 (65.9%)
Outpatient (n; %)	327 (34.1%)
ERCP performed in emergency (n; %)	40 (4.2%)
Repeated ERCP vs. first-time ERCP	27 (2.8%)
USE OF STATINS (n; %)	
No statin consumption	497 (51.9%)
Statin consumption	461 (48.1%)
Incidence of post-ERCP pancreatitis/hyperamylasemia (n; %)	161 (16.8%)
Hyperamylasemia	107 (11.2%)
PEP	54 (5.6%)
Severe pancreatitis	6 (0.6%)

In the univariate analysis, while ethnicity (Jewish) (*p* = 0.014) and incidence of pancreatitis/hyperamylasemia (*p* < 0.001) were highly significant among the statins treated group, age was borderline significant (Table 2). Specifically, statistical significance was achieved both for PEP (8.4 vs. 2.6%, *p* < 0.001) and hyperamylasemia (14.1 vs. 8.1%, *p* = 0.003), for non-consumers and consumers of statins, respectively. The logistic regression analysis (Table 3), after correcting for all potential confounding factors, demonstrated that chronic use of statins is a protective factor in preventing development of PEP/hyperamylasemia (OR 0.436 [95%CI 0.303–0.627], *p* < 0.001); Particularly, the PEP OR was of 0.318 [95%CI 0.169–0.597], *p* < 0.001 and the hyperamylasemia OR was of 0.565 [95%CI 0.372–0.859], *p* = 0.0008. No significant predictor could be found for the risk of developing severe PEP.

**Table 2:** Univariate analysis showing comparison between consumers and non-consumers of statins.

**Variable**	**No use of statins (*n* = 497)**	**Use of statins (*n* = 459)**	**Statistical significance**
Age	60.35 ± 22.30	63.81 ± 19.77	0.051
Gender			1.000
Male	207 (41.6%)	192 (41.8%)	
Female	290 (58.4%)	267 (58.2%)	
Ethnicity			**0.014**
Non-Jew	55 (11.1%)	76 (16.6%)	
Jew	442 (88.9%)	383 (83.4%)	
Impatient vs. outpatient	327 (65.8%)	303 (66.0%)	0.946
Emergency	18 (3.6%)	22 (4.8%)	0.420
Repeated ERCP vs. first-time ERCP	12 (2.4%)	15 (3.3%)	0.426
Pancreatitis/hyperamylasemia	112 (22.5%)	49 (10.7%)	**<0.001**
Pancreatitis	42 (8.4%)	12 (2.6%)	**<0.001**
Hyperamylasemia	70 (14.1%)	37 (8.1%)	**0.003**
Severe pancreatitis	5 (1.0%)	1 (0.2%)	0.220

*Bold values indicates statistically significant*.

**Table 3:** Predictors of developing post-ERCP pancreatitis and/or hyperamylasemia at the logistic regression.

**Source**	**Value**	**Standard error**	**Wald Chi-Square**	**Pr > Chi2**	**Wald lower bound (95%)**	**Wald upper bound (95%)**	**Odds ratio**	**Odds ratio lower bound (95%)**	**Odds ratio upper bound (95%)**
**PEP/HYPERAMYLASEMIA**									
Intercept	−0.907	0.297	9.297	**0.002**	−1.489	−0.324			
Age	−0.004	0.004	1.221	0.269	−0.012	0.003	0.996	0.988	1.003
Origin (non-Jew vs. Jew)	−0.392	0.290	1.823	0.177	−0.961	0.177	0.676	0.383	1.194
Gender (male vs. female)	0.023	0.179	0.016	0.899	−0.328	0.373	1.023	0.720	1.453
Use of statins	−0.830	0.186	20.015	**<0.001**	−1.194	−0.466	0.436	0.303	0.627
Inpatient vs. outpatient	0.008	0.188	0.002	0.966	−0.361	0.377	1.008	0.697	1.458
Emergency	−0.081	0.452	0.032	0.858	−0.966	0.805	0.922	0.380	2.236
Repeated ERCP vs. first-time ERCP	−2.379	1.452	2.684	0.101	−5.225	0.467	0.093	0.005	1.595
**PEP**									
Intercept	−2.028	0.454	19.922	**<0.001**	−2.918	−1.137			
Age	−0.007	0.006	1.181	0.277	−0.019	0.005	0.993	0.982	1.005
Origin (non-Jew vs. Jew)	−0.531	0.498	1.135	0.287	−1.507	0.446	0.588	0.221	1.561
Gender (male vs. female)	0.050	0.281	0.032	0.859	−0.500	0.600	1.051	0.606	1.822
Use of statins	−1.147	0.322	12.658	**<0.001**	−1.779	−0.515	0.318	0.169	0.597
Inpatient vs. outpatient	0.209	0.305	0.470	0.493	−0.389	0.807	1.233	0.678	2.241
Emergency	0.012	0.683	0.000	0.986	−1.326	1.350	1.012	0.266	3.858
Repeated ERCP vs. first-time ERCP	−1.087	1.445	0.566	0.452	−3.920	1.745	0.337	0.020	5.726
**HYPERAMYLASEMIA**									
Intercept	−1.530	0.350	19.140	**<0.0001**	−2.215	−0.844			
Age	−0.003	0.005	0.288	0.592	−0.012	0.007	0.997	0.988	1.007
Origin (non-Jew vs. Jew)	−0.240	0.329	0.531	0.466	−0.884	0.405	0.787	0.413	1.499
Gender (male vs. female)	0.006	0.209	0.001	0.975	−0.404	0.417	1.007	0.668	1.517
Use of statins	−0.571	0.214	7.145	**0.008**	−0.990	−0.152	0.565	0.372	0.859
Inpatient vs. outpatient	−0.102	0.218	0.221	0.638	−0.529	0.324	0.903	0.589	1.383
Emergency	0.004	0.523	0.000	0.994	−1.021	1.029	1.004	0.360	2.799
Repeated ERCP vs. first-time ERCP	−1.930	1.445	1.785	0.182	−4.762	0.902	0.145	0.009	2.464
**SEVERE PANCREATITIS**									
Intercept	−3.268	1.673	3.816	0.051	−6.546	0.011			
Age	0.013	0.019	0.472	0.492	−0.025	0.051	1.013	0.976	1.053
Origin (non-Jew vs. Jew)	−0.462	1.691	0.075	0.784	−3.777	2.852	0.630	0.023	17.317
Gender (male vs. female)	0.541	0.787	0.472	0.492	−1.001	2.083	1.717	0.367	8.025
Use of statins	−0.172	0.967	0.032	0.859	−2.069	1.724	0.842	0.126	5.606
Inpatient vs. outpatient	0.802	0.958	0.700	0.403	−1.076	2.679	2.229	0.341	14.571
Emergency	0.201	2.058	0.010	0.922	−3.833	4.235	1.223	0.022	69.077

*Bold values indicates Statistically significant*.

## Discussion

In the current study, chronic use of statins (>6 months) was found to be a protective factor against the development of PEP, however, it did not significantly affect the severity of pancreatitis.

PEP/hyperamylasemia is a major complication of the ERCP procedure (Szary and Al-Kawas, 2013). Therefore, many efforts have been made in order to decrease the rates of such complications (Elmunzer et al., 2008; Zheng et al., 2008). Prophylactic pancreatic stent placement (PSP) seems able to prevent PEP (risk ratio or RR = 0.28 [95%CI 0.18–0.42]; Hu et al., 2016; Vadalà di Prampero et al., 2016). Another study has found that PSP can significantly decrease the risk of PEP in high risk patients (OR = 0.44, [95%CI 0.24–0.81]) and almost always prevents severe cases (Andriulli et al., 2007).

Concerning the prophylactic administration of drugs, NSAIDs such as indomethacin and diclofenac represent two valid strategies, as a recent meta-analysis of 17 trials, pooling 4,741 patients, has shown a significantly decreased RR of PEP (RR = 0.60 [95%CI 0.46–0.78]; Patai et al., 2017). Bolus-administered somatostatin has shown similar effects (RR = 0.63 [95%CI 0.40–0.98]; Hu et al., 2016). Inconsistent results were reported regarding the use of corticosteroids as a preventive therapy for PEP. One study has found that the incidence of PEP in the corticosteroid treated group is around 4.6%, whereas in the control group it was 7.4–9.1% (Weiner et al., 1995). Nevertheless, in other studies, corticosteroids failed to reduce the risk of PEP (Bai et al., 2008; Zheng et al., 2008).

Lee et al. (2017) carried out a historical cohort study recruiting adult patients with acute pancreatitis, matching patients on (*n* = 110) and off (*n* = 210) statins based on a propensity score in order to simulate a randomized controlled trial. Authors found that patients on statins were less likely to develop multisystem organ failure, severe acute pancreatitis and necrosis. Similarly, a retrospective cohort study based on data from an integrated healthcare system in southern California performed by Wu et al. (2015) found that simvastatin use reduced significantly the emergence of acute pancreatitis (crude incidence rate ratio or RR = 0.626 [95%CI 0.588–0.668], adjusted RR = 0.29 [95%CI 0.27–0.31]). On the other hand, Kuoppala et al. (2015) performed a registry-based case-control study based on 4,376 patients hospitalized for acute pancreatitis age- and sex-matched with 19,859 randomly selected controls from the adult population of Finland, with 19% cases and 13% controls having been exposed to statins. Authors found that statin use was associated with an increased incidence rate of acute pancreatitis (OR 1.25 [95%CI 1.13–1.39]), especially during the first year of use. This was demonstrated both among current (OR of 1.37 [95%CI 0.94–2.00] for at most 3 months of use and OR of 1.32 [95%CI 1.07–1.63] for 4–12 months of use) and former users (OR 1.64 [95%CI 1.33–2.03]). Shiu et al. (2015) enrolled 31 patients on statins matched with 63 controls and found that statins had no beneficial effects on the clinical outcomes of patients with a first onset acute pancreatitis, while having a positive effect on the computed tomography severity index.

To the best of our knowledge, this is one the first studies to prove that chronic statin use reduces the risk of emergence of PEP. The only group to perform a similar investigation is of de Madaria et al. (Martinez-Moneo et al., 2017). Authors recruited a sample of 708 patients (median age of 70 years, 53.5% men, 29.5% on statins), and computed an incidence of PEP of 1.9% and of 5.8% among the consumers and non-consumers of statins, respectively. In the multivariate analysis, lack of statin consumption was shown to be an independent risk factor for the development of PEP (OR 3.56 [95%CI 1.19–10.68], *p*-0.023). A potential plausible mechanism by which statins might prevent PEP is their reported anti-inflammatory effects (Blake and Ridker, 2000). Indeed, statins can modulate the synthesis of isoprenoids thus affecting the pathways involving endothelial nitric oxide synthase (Endres et al., 1998). Additionally, statins were found to have immunomodulatory effects on T-cells, inducing the secretion of anti-inflammatory cytokines such as IL-10 and transforming growth factor (TGF)-beta (Youssef et al., 2002). Furthermore, several studies have shown the capacity of statins to reduce CRP levels in patients with acute coronary disease and type II diabetes, despite lack of changes in cholesterol levels (Kluft et al., 1999; Strandberg et al., 1999). It was also found that adherence to statins is associated with lower risk of developing rheumatoid arthritis (Chodick et al., 2010). Therefore, the reduced risk of PEP found in our study might be attributed to the heterogeneous immunomodulatory effects of statins as found in the previous studies.

However, some drawbacks affect our investigation, the major limitation being the study design i.e., a retrospective database-based study as opposed to a randomized controlled trial (RCT) and missing data concerning levels of triglycerides.

In conclusion, different approaches with varying efficacies may be used in order to prevent the emergence of PEP/hyperamylasemia. Our study seems to suggest the possibility of exploiting statins as pharmacopreventative agents. However, despite the strengths of the study, prospective randomized trials are required to confirm our findings, also using different ethnicities.

## Author contributions

All authors contributed to the research. MM, AW, MA, and EG were involved in conception and design. NB and AW performed statistical analysis. DW, DL, MM, and JW were involved in data collection. AW, JW, and HA wrote the draft and final manuscript. EG and HW performed critical review and revision.

### Conflict of interest statement

The authors declare that the research was conducted in the absence of any commercial or financial relationships that could be construed as a potential conflict of interest.
